# Health Risks and Contamination Levels of Heavy Metals in Dusts from Parks and Squares of an Industrial City in Semi-Arid Area of China

**DOI:** 10.3390/ijerph14080886

**Published:** 2017-08-07

**Authors:** Xiufeng Han, Xinwei Lu, Qinggeletu, Yongfu Wu

**Affiliations:** 1 Department of Environmental Science, School of Geography and Tourism, Shaanxi Normal University, Xi’an 710119, China; hxf9620@126.com (X.H.); yongfu2006@126.com (Y.W.); 2 College of Resources and Environment, Baotou Normal College, Science and Technology University of Inner Mongolia, Baotou 014030, China; 3 Department of Geography, North heavy industry No.3 Middle School, Baotou 014030, China; kexm2010@163.com

**Keywords:** heavy metal, dust, contamination level, health risk assessment, Baotou city

## Abstract

The contamination characteristics and health risk of barium (Ba), cobalt (Co), chromium (Cr), copper (Cu), manganese (Mn), nickel (Ni), lead (Pb), vanadium (V), zinc (Zn), arsenic (As), mercury (Hg), and cadmium (Cd) in samples of dust gathered from squares and parks of Baotou city, an industrial city situated in a semi-arid location of the northwest China were investigated. The contents of Ba, Co, Cr, Cu, Mn, Ni, V, Pb, and Zn in the collected dust samples were determined using X-ray fluorescence spectrometry, while the contents of As and Hg in the dust were investigated by use of the ICP-MS. Further, cadmium was quantified through the atomic absorption spectrometry. Levels of contamination of heavy metals analyzed in the dust samples were evaluated using the Geo-Accumulation index (*I*_geo_) as well as through a Pollution Load Index (*PLI*). Their health risks to children and adults were evaluated based on the US EPA model of health risk. The findings portrayed that the mean concentrations of Ba, Co Cr, Cu, Pb, V, Cd, and Hg were elevated as compared with their local soil background values. Mean values of *I*_geo_ illustrate the order of Co > Cr> Cd > Hg > Pb > Cu > Ba > V > Ni > Mn > Zn > As. It was evident that dusts from the parks and squares were “unpolluted” to “moderately polluted”. Assessment of health risk depicts that ingestion is the foremost route of exposure in regard to the heavy metals, then the dermal adsorption follows. Hg exposure from dust might also set impending health threats to children. Besides, the cancer risks of Co, Cr, Ni, Cd, and As are considered to be within the presently tolerable range.

## 1. Introduction

Dusts found on impermeable urban surfaces has become among the most significant concerns in management of urban environment [1]. Urban dusts worldwide have elevated concentrations of toxic metals [2,3,4,5], establishing a latent danger to people’s health as well as threats to the ecological system [6,7,8]. Continuous deposition of heavy metals in urban areas may also act as a secondary source of pollution. The most vulnerable people who are affected by these dusts are the elderly as well as children. This is because their immune systems are immature and age-compromised, respectively. They also experience the non-premeditated ingestion of considerable amounts of dusts via hand-to-mouth passageways, and thus are more susceptible to contaminated dusts [1,9,10]. For example, high levels of heavy metals might build up in our bodies’ fatty tissues, thereby affecting our Central Nervous System (CNS). They might also be set down in our bodies’ circulatory system, which might lead to disruption of the usual performance of our internal organs. Besides, they might also operate as co-factors in causing other diseases [11,12,13,14,15]. Lead (Pb) is associated with lead levels in children’s blood, affecting central nervous and skeletal systems [16,17]. So, by ingestion, dermal contact, and inhalation, urban residents and tourists may be exposed to more heavy metals, especially in parks and squares [18].

Urban parks, as well as squares, are utilized for leisure activities or sporting and other recreational activities. They are the most important places where outdoor activities can be held for urban residents. In China, with improvements in lifestyle, residents have started to pay attention to their health. So, many old people and children throng into the squares and parks to carry out various activities such as square dancing. Therefore, environmental quality of urban parks and squares directly affect the health of these residents as well as residents living around the parks and squares. Dusts from these areas can get through people’s bodies by re-suspension–inhalation, hand–mouth ingestion, or through dermal contact, thereby endangering people’s health. Thus, it is important to explore contamination levels caused by dusts from the parks and squares and the resultant health effects on people living around or using these locations or spaces.

Whereas a majority of previous studies were conducted on effects of street dusts in developed nations or in megacities [19,20,21,22,23,24,25], there has not been distinct study carried on dust contamination brought about by heavy metals in public squares and parks, particularly in rapidly industrializing or urbanizing cities of the semi-arid area of Western China.

Baotou city is a young and medium-sized industrial city situated in the semi-arid region of northwest China. Just as with other industrialized cities, Baotou tends to also experience a lot of challenges related to the environment, especially caused by adverse conditions of the environment, poor planning of the city, as well as quick advancement of the city, especially after the implementation of the Chinese Great Western Development Policy in 1990s. To our knowledge, information on heavy metal contamination in dusts collected from the city’s parks and squares is lacking. Therefore, this study was carried out to determine dusts with heavy metal contamination collected from the urban parks and squares of Baotou so as to assess dust contamination levels and respective health risks of these heavy metals on residents and those using such spaces. The findings of this study could offer basic information for regulators and urban square/park users.

## 2. Materials and Methods

### 2.1. Background of Study Area

**Figure 1:**
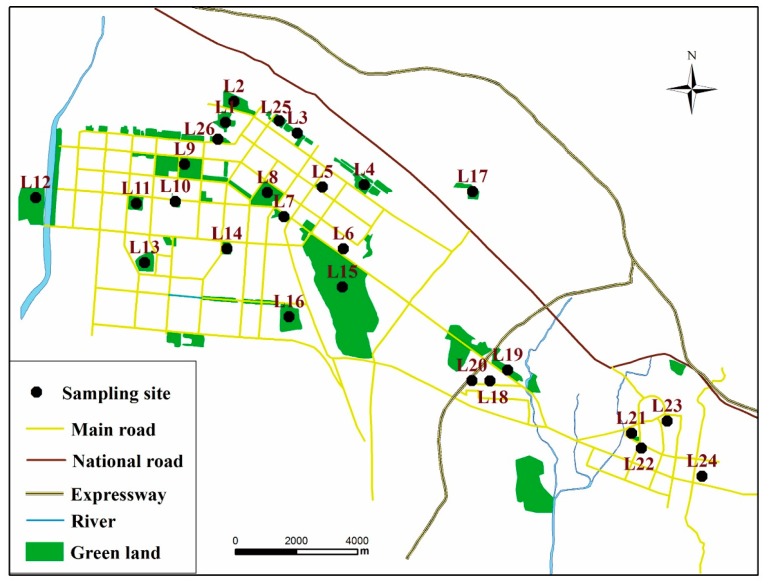
Study area and sampling sites in Baotou, China.

Baotou (109°15′–110°26′ E, 40°15′–42°43′ N) is the principal industrial city in the Inner Mongolia sovereign area. It is situated in the Tumochuan and Hetao Plain, with the Yellow River to its South and Mongolia to its North (Figure 1). Baotou city’s climate is a distinctive semi-arid temperate continental monsoon type of climate. The yearly average temperature is 6.5 °C, with a yearly average precipitation between 240 and 400 mm. Moreover, the city experiences yearly evaporation capacity of about 1940–2340 mm. The city’s urban coverage includes four districts, which are Kundulun District, Qingshan District, Jiuyuan District, and Donghe District. The city’s average area and the urban area are estimated to be 27,768 and 2965 km^2^, respectively, with a resident population of 2,766,200 people in 2013. Being an industrial base and a rare earth industrial center, Baotou is famous for its main industries such as rare earth industry, iron and steel manufacturing, coal-fired power generating, heavy duty vehicle, aluminum smelter, metallurgy, machinery manufacturing, and dairy. Baotou is a famous industrial city with iron and steel, rare earth, and known as the “national garden city”. Residents can find fitness and recreation parks and squares around their houses. According to statistics, Baotou has 21 large open parks, 39 squares with more than 10,000 m^2^. The total area of parks is approximately 18.66 km^2^. The 26 squares and public parks that are examined in the present study for the main sites in Baotou, and are surrounded with residential areas, thus offering a wide spectrum of activities such as leisure, sporting, or recreation.

### 2.2. Sampling and Analytical Procedures

Samples of dust were acquired from the 26 parks and squares in four districts of Baotou urban area, including Kundulun District, Qingshan District, Jiuyuan District, and Donghe District (Figure 1; Supplementary Materials Table S1). From each park/square, 15 to 20 sampling sites were uniformly selected according to their sizes. From every site, a composite of dust of about 300–500 g was collected in through sweeping with clean plastic brushes and using dust pans to collect the samples in May 2014. Care was observed in reducing the interruption of the small particles. Besides, any noticeable unrelated matter like stones and leaves were also taken away from the samples during the sampling stage. The collected samples were then stored up in self-preserved polyethylene bags, labeled, and then taken to the laboratory. After they were air-dried at room temperature, the samples were sifted using a 1.0 mm mesh nylon sieve so as to take away the large plant parts as well as irrelevant gravel-sized materials in the samples. About 50 g of each of the dried samples were split through the quartering method. They were then ground using a vibration mill, and sifted further using a 75 μm nylon mesh. All the procedures of handling the samples were observed, ensuring that there is no contact with the metals. This was observed to evade the likelihood of cross-contamination of the samples.

A total of 4.0 g of milled dust samples along with 2.0 g of boric acid were then measured out, put in the mold, and then compressed into a 32 mm diameter pellet under 30 t pressures [5]. Concentrations of barium, cobalt, chromium, copper, manganese, nickel, lead, vanadium, and zinc in every sample were directly quantified through the wavelength dispersive X–ray fluorescence spectrometry (XRF, PW-2403, PANalytical, Almelo, The Netherlands) apparatus, with a detection limit of 0.1 mg/kg [5]. The concentrations of arsenic and mercury in the dust sample were determined by ICP-MS (X Series 2, DG-03, ThermoFisher Scientific, Waltham, MA, USA), with a detection limit of 0.1 mg/kg for arsenic and 0.004 mg/kg for mercury. The concentration of cadmium in the dust samples was determined using graphite furnace atomic absorption spectrometry (Avanta YX-06, GBC, Melbourne, Australia) after the HNO_3_-HF-HClO_4_ mixed acid digestion, with a detection limit of 0.02 mg/kg for cadmium. Standard reference soil samples (GSS-4, GSS-2) (Institute of Geophysical and Geochemical Prospecting, Langfang, China), blank samples, and duplicate samples were then concurrently investigated to offer quality accuracy as well as quality control in the experiments [5,26,27]. The analytical precision, measured as relative standard deviation, was routinely 3–5%. Accuracy of the analyses was checked using standard and duplicate samples. The quality control gave good precision (S.D. < 5%).

### 2.3. Pollution Assessment Methods

Geo-Accumulation Index (*I*_geo_) along with Pollution Load Index (*PLI*) were utilized in the present research to evaluate the levels of contamination of heavy metals found in the analyzed dust samples. *I*_geo_ of each heavy metal was measured through the following equation [27]

(1) $$I_{\mathrm{geo}}=\log_{2}\frac{C_{i}}{1.5B_{i}}$$

where *C_i_* is the calculated heavy metal’s *i* concentration in the sample; *B_i_* is the heavy metals’ *i* geochemical background value. For this present study, *B_i_* is the background value of local soil [28]. The constant 1.5 is brought in to bring down the consequence of likely deviation in the background values. The *I*_geo_ for every heavy metal was computed and categorized as: “uncontaminated” (*I*_geo_ ≤ 0); “uncontaminated to moderately contaminated” (0 < *I*_geo_ ≤ 1); “moderately contaminated” (1 < *I*_geo_ ≤ 2); “moderately to heavily contaminated” (2 < *I*_geo_ ≤ 3); “heavily contaminated” (3 < *I*_geo_ ≤ 4); “heavily to extremely contaminated” (4 < *I*_geo_ ≤ 5); “extremely contaminated” (*I*_geo_ ≥ 5) [27].

*PLI*, which is an integrated pollution index was also utilized in assessing the levels of pollution of heavy metals at the selected sites [10,14]. This was calculated using the formula

(2) $$PLI=\sqrt[{n}]{\overset{n}{\underset{i=1}{\Pi }}C_{i}/B_{i}}$$

where *C_i_* is the heavy metal’s *i* concentration in the samples of dust; *B_i_* is the heavy metal’s *i* background value. The result of *PLI* = 0 means “background concentration”, 0 < *PLI* < 1 means “unpolluted”, 1 < *PLI* < 2 indicates “unpolluted to moderately polluted”, 2 < PLI < 3 means “moderately polluted”, 3 < *PLI* < 4 signifies “moderately to highly polluted”, 4 < *PLI* < 5 depicts “highly polluted” whereas *PLI* > 5 corresponds to “very highly polluted” [15,29].

### 2.4. Health Risk Assessment

In this study, the model that was used to assess health risk of collected samples of dusts containing heavy metals was derived from a model advanced by the United States Environmental Protection Agency [30], as well as the Dutch National Institute of Public Health and Environmental Protection [31]. It was widely used by other authors in the literature [32,33,34,35,36]. Residential exposure of heavy metals in dusts might take place out of three major routes: (a) ingestion of dust particles (*D*_ing_); (b) inhalation of dust particles via the mouth or nose (*D*_inh_); (c) dermal contact (*D*_dermal_), and via inhalation of vapors (*D*_vapor_). The exposure dose of heavy metals measured in the dust from every possibility was measured through the use of these Equations (3)–(5) [30,37]. Exposure of mercury might also take place through vapor inhalation, that might be expressed by the Equation (6). For carcinogens, the Lifetime Average Daily Dose (*LADD*) (inhalation exposure route for cobalt, chromium, nickel, and cadmium; inhalation, ingestion as well as dermal adsorption exposure route for arsenic) was utilized in the evaluation of cancer risk [30,38] then calculated by Equation (7).

(3) $$D_{ing}=C\times \frac{IngR\times EF\times ED}{BW\times AT}/10^{6}$$

(4) $$D_{inh}=C\times \frac{InhR\times EF\times ED}{PEF\times BW\times AT}$$

(5) $$D_{dermal}=C\times \frac{SL\times SA\times ABS\times EF\times ED}{BW\times AT}/10^{6}$$

(6) $$D_{vapour}=C\times \frac{InhR\times EF\times ED}{VF\times BW\times AT}$$

(7) $$LADD=\frac{C\times EF}{AT\times PEF}\times (\frac{CR_{child}\times ED_{child}}{BW_{child}}+\frac{CR_{adult}\times ED_{adult}}{BW_{adult}})$$

where *C* is the concentration of heavy metal in the dust (exposure-point concentration), together with the values for the exposure factors, which are taken to produce an approximate of the “reasonable maximum exposure” [37]. This is the upper limit of the 95% confidence interval for the mean (95% UCL); *CR* is the contact or adsorption rate (that is ingestion (*CR* = *IngR*), inhalation (*CR* = *InhR*), and dermal adsorption (*CR* = *SA* × *SL* × *ABS*) rates) [32]. The meanings and values of other parameters are listed in Supplementary Materials Table S2.

The calculated doses for every element and pathways of exposure are consequently divided up through the corresponding Reference Dose (*RfD*) to produce a Hazard Quotient (HQ) (or non-cancer risk), while for the carcinogens, the dose is proliferated through the corresponding Slope Factor (SF) to yield a level of risk of cancer. The Hazard Index (HI) then becomes the sum of HQ. If HI < 1, it is considered that “no significant risk” of non-carcinogenic effects exists. However, when HI > 1, then there is a probability of non-carcinogenic effects occurring, with a possibility which seems to improve as the value of HI increases too [38]. The carcinogenic danger is, therefore, the possibility of a person acquiring any kind of cancer from lifetime contact with the carcinogenic risks. The conventional or allowable risk for regulatory reasons is usually in the range of 10^−6^–10^−4^ [14,15,32]. For the present study, Hazard Index methodologies as well as cancer risk methodologies were utilized in assessing the health risks of metal exposure to dusts in the parks as well as from the squares of Baotou.

## 3. Results and Discussion

### 3.1. Heavy Metal Concentration in Dust of Park and Square

Concentration of heavy metals in dust samples collected from 26 parks and squares of Baotou, and the background values of local soil [28] were summarized in Table 1. It is observable from Table 1 that the mean concentrations of manganese, nickel, zinc, and arsenic were less than or close to the corresponding background values of local soil [28]. The mean concentrations of barium, cobalt, chromium, copper, lead, vanadium, cadmium, and mercury in the dust samples were respectively 1.2-, 5.4-, 2.7-, 1.4-, 1.9-, 1.1-, 2.4-, and 2.6-times of the background value of local soil [28], indicating that the dusts were polluted by these metals, especially cobalt, chromium, cadmium, and mercury. The Coefficients of Variation (CVs) of cobalt, copper, manganese, lead, zinc, cadmium, and mercury in the dust are relatively larger than 20%. Particularly, the CVs for copper, zinc, and mercury in the dust are > 40%, which depicts that copper, zinc, and mercury in the dust of the parks and squares in Baotou city are greatly disturbed by human activities.

Due to the surrounding environment and the construction time of the parks and squares, there exist great variations in the accumulation of heavy metals in these areas. Highest values of barium, chromium, manganese, lead, and vanadium were found in Baogang Park (L12), West Industrial Park of Baotou city—in which a steel smelting plant and thermal power plants are located—the quantities derived from this location were 845.8, 379.8, 1148.4, 68.9, 108.7 and 88.7 mg/kg, respectively. The highest value of cobalt was located in First Machinery Factory Park (L4), which was found to be 11.3 times more than the background value of local soil. Some studies indicated that cobalt is usually utilized as alloying additions in machinery industries. Cobalt is, therefore, widely utilized in producing stainless steels and alloys [39]. It is also utilized in jets, gas turbines, and other such devices that operate under high temperatures. Besides, cobalt compounds might also be utilized as catalysts and porcelain glaze [39]. Therefore, in Baotou city, the high value of cobalt may be related to the distribution of machinery manufacturing plant in the city. There are two high-value point of nickel (32.4 mg/kg) in the study area, that is, the First Machinery Factory Park (L4) and in the Donghe District government square (L24). The highest value (2.8-times the background value) of copper is situated at Donghe District government square (L24) which experiences heavy traffic. The highest values (1.6-times the background value) of zinc and arsenic are found at the Baotou Paradise (L13) which is densely populated. The highest value of cadmium is found in Kundulun government square (L10), which is a commercial center experiencing heavy traffic, and the secondary maximum of cadmium is found in Baogang Park (L12). The highest value of mercury is found at Weapon Park (L25), which is a neighborhood with a coal-fired power plant.

Lowest values of copper, manganese, lead, vanadium, zinc, and cadmium appeared in Saihantala Park (L15), with highest greening. Lowest value of barium, cobalt, chromium, nickel, arsenic, and mercury were found in the Siji Square (L26), Aerding plant garden (L9), Renmin Park (L21), and Jinglin Park (L2), respectively.

In general, the mean contents of barium, cobalt, chromium, cadmium, manganese, lead, vanadium, arsenic, and mercury in dust samples collected from the parks and squares of Kundulun District and Qingshan District were higher than those of other areas. On the other hand, the concentrations of copper, nickel, and zinc in dust samples collected from Donghe District were higher than those of other areas, which may be related to human activities in the area. Steel plant, coal-fired power plants, metallurgical industry, commercial center, and traffic density are mainly located at the Kundulun District and Qingshan District, while Donghe District is the commercial center and aluminum production base. The spatial variability of heavy metals in dust samples from the parks and squares of Baotou city implies their sources. Based on the foregoing analyses, we can conclude that barium, chromium, lead, and vanadium mainly derived from the emissions of steel smelting plant and thermal power plant; cobalt was mainly from machinery manufacturing; cadmium has the mixed sources of steel smelting, thermal power plant, and traffic exhaust; copper and zinc primarily originated from traffic emissions and nature; mercury was principally from coal-fired power plant; and manganese, nickel, and arsenic were mainly from natural source (local soil).

### 3.2. Metal Pollution Assessment Results

**Figure 2:**
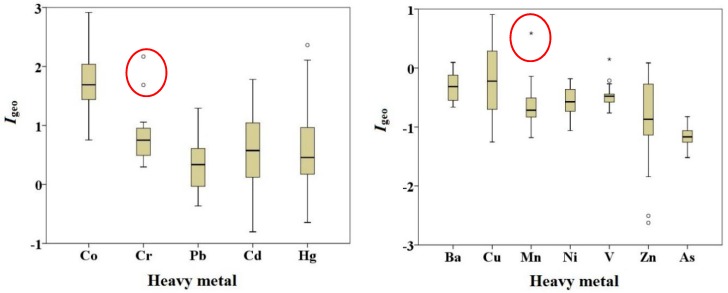
Box-plot of *I*_geo_ for heavy metals in dust samples collected from parks and squares of Baotou (* extreme outlier; ° mid outlier).

The calculated results of *I*_geo_ for all the analyzed heavy metals in dust samples collected from squares and parks of Baotou city are shown in Figure 2. It can be seen from Figure 2, the *I*_geo_ values of barium, cobalt, chromium, copper, manganese, nickel, lead, vanadium, zinc, cadmium, arsenic, and mercury range from −0.66 to 0.10, 0.75 to 2.92, 0.30 to 2.17, −1.25 to 0.91, −1.18 to 0.59, −1.06 to −0.18, −0.36 to 1.29, −0.76 to 0.15, −2.62 to 0.09, −0.80 to 1.78, −1.52 to −0.82, and −0.64 to 2.36, with an average of −0.32, 1.76, 0.80, −0.22, −0.64, −0.57, 0.30, −0.47, −0.89, 0.60, −1.16, and 0.58, respectively. The calculated results of *I*_geo_ of heavy metals decrease in the order of Co > Cr > Cd > Hg > Pb > Cu > Ba > V > Ni > Mn > Zn > As. The mean *I*_geo_ values of cobalt, chromium, lead, cadmium, and mercury are > 0—most notably, the highest values of copper, chromium, and mercury are > 2—indicating the dusts from parks and squares of Baotou city are polluted by these heavy metals. The *I*_geo_ values of nickel and arsenic in all dust samples are < 0, indicating an “uncontaminated” status.

The mean *I*_geo_ and 69% *I*_geo_ of cobalt are between 1 and 2, demonstrating “moderately contaminated”, while 27% *I*_geo_ of cobalt is in 2–3, depicting “moderately to heavily contaminated”. The mean *I*_geo_ and 81% *I*_geo_ of chromium are in 0–1, illustrating “uncontaminated to moderately contaminated”, while 15% *I*_geo_ of chromium is between 1 and 2, indicating “moderately contaminated”. The mean *I*_geo_ of lead and cadmium, 69% *I*_geo_ of lead and 62% *I*_geo_ of cadmium are between 0 and 1 demonstrating “uncontaminated to moderately contaminated”, 4% *I*_geo_ of lead and 27% *I*_geo_ of cadmium are in 1–2 demonstrating “moderately contaminated”, while 27% *I*_geo_ of lead and 12% *I*_geo_ of cadmium are <0 illustrating “uncontaminated”. The mean *I*_geo_ and 62% *I*_geo_ of copper are <0, illustrating “uncontaminated”, while 38% *I*_geo_ of copper is in 0–1 indicating “uncontaminated to moderately contaminated”. Mercury has a large pollution range, *I*_geo_ values in 19% samples are < 0, in 58% samples are between 0 and 1, in 15% samples are between 1 and 2 and in 8% samples are between 2 and 3, indicating that mercury in the dusts is mainly “uncontaminated to moderately contaminated” and in part of other samples, presenting a “heavily contaminated” status. The *I*_geo_ values of barium, manganese, vanadium, and zinc in most of the samples are less than 0, depicting an “uncontaminated” status.

**Figure 3:**
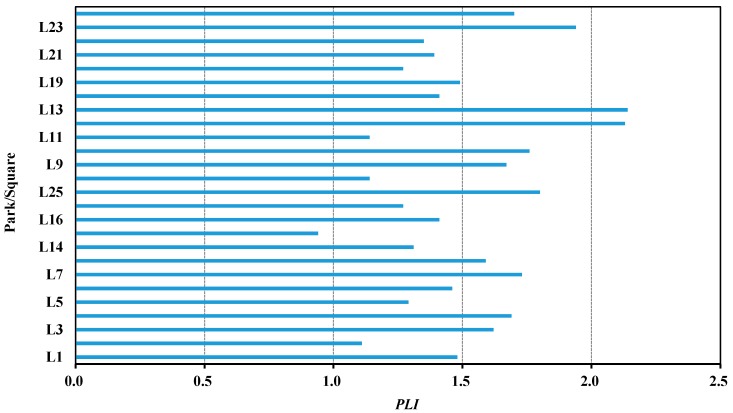
*PLI* values of heavy metals in dust samples collected from parks and squares of Baotou.

The calculated *PLI* values of heavy metals in the dust samples acquired from the parks and squares of Baotou city are illustrated in Figure 3. The *PLI* values of metals in all dust samples were found to range from 1.00 to 2.14, with an average of 1.57. This shows that the parks and squares of Baotou city were polluted with these heavy metals in various intensities. *PLI* values in dusts from Baogang Park (L12) and Baotou Paradise (L13) were in the 2–3 range, indicating “moderately polluted”, while the *PLI* values from other parks and squares were in the 1–2 range, indicating “unpolluted to moderately polluted”. The highest value of *PLI* was captured in the Baotou Paradise (L13), whereas the minimum value of *PLI* was found in the Saihantala Park (L15). The *PLI* values in the four districts decreased from Kundulun District (1.73) > Donghe District (1.58) > Qingshan District (1.40) > Jiuyuan District (1.39). Kundulun District depicted the highest value of *PLI* in the four districts, which was mainly due to the coal-fired power plant, steel plant, metallurgical industry, and commercial center locations experiencing heavy traffic.

### 3.3. Health Risk Assessment of Heavy Metal Exposure to Dust

The findings of health risk assessment show that, for non-cancer consequences, in regards to children, intake of dust particles seems to be the major way of exposure to the metals in dust, causing greater health risks. Dermal adsorption apart from effects emanating from copper and manganese (Supplementary Materials Table S3) is the subsequent health risk. In essence, the relative contributor of ingestion exposure to hazard index (HI) ranges from 78.1% for vanadium and cadmium to 99.1% for copper, showing that the ingestion of dust particles are the largest cause of health risk. Comparable deductions have also been illustrated in the literature [13,14,15,32]. HQs of copper, nickel, lead, vanadium, zinc, cadmium, arsenic, and mercury for dust particle ingestion are in the range of 4–5 orders of magnitude greater than for inhalation of dust particles (Supplementary Materials Table S3). Thus, inhalation of re-suspended dust particles via the nose or mouth is nearly insignificant as compared to the other ways of contact. Nonetheless, inhalation of mercury vapor as the fourth exposure pathway to dust is significant, which accounts for the major exposure to dust in the squares and parks of Baotou city. HI for the analyzed metals in dust from Baotou decrease in the sequence of Cr > As > Mn > V > Pb > Ba > Co > Hg > Ni > Cu > Cd > Zn. Chromium (0.5) and arsenic (0.2) depict HI higher than 0.1, demonstrating that there is a slight undesirable health risk owing to the dust. Nevertheless, the heavy metals might also build up in the body for long, instigating non-cancer effects for chromium and arsenic.

Health risks are higher in children as compared to adults, meaning that the children are likely to face greater dangers as a result of exposure to the dust. For barium, cobalt, copper, manganese, nickel, lead, and zinc, the ingestion of these dust particles tend to be the major cause of exposure to the heavy metals, resulting in a higher health risk. Conversely, chromium, vanadium, cadmium, and arsenic are related to dermal adsorption which tends to be the main exposure way for the heavy metals found in dust particles. Inhalation of mercury vapor is the fourth exposure route to dust. HIs for the assessed metals to adults also showed a decrease in the order of Cr > As > V > Mn > Ba > Pb > Hg > Co > Cd > Ni > Cu > Zn. The HI values for all metals owing to exposure to dust for this study were found to be within the safe levels that are proposed by USEPA [37].

For cancer risk, the results show that risks for cobalt, chromium, nickel, cadmium, and arsenic as a result of exposure to dust in the Baotou city decreased in the sequence of Cr > Co > As > Ni > Cd. Levels of risks for cancer for cobalt, chromium, nickel, cadmium, and arsenic were lower than the tolerable range (10^−6^–10^−4^) above which the environmental and regulatory agencies perceive as an unacceptable risk. Thus, the cancer risk from exposure to cobalt, chromium, nickel, cadmium, and arsenic can be said to be low. The actual risks of heavy metals exposure in the dusts from parks and squares of Baotou city to local people should be conducted by human biomonitoring. Human biomonitoring can measure the internal dose of a chemical resulting from integrated exposures from all exposure routes [40]. In human biomonitoring, blood, urine, and/or hair samples of local people can be collected and the contents of heavy metals in these types samples can reflect the actual exposure dose of heavy metals [40,41,42,43,44]. This is the future work of our group.

## 4. Conclusions

Considering the levels of concentrations, contamination and the heavy metals’ health risks for barium, cobalt, chromium, copper, manganese, nickel, lead, vanadium, zinc, cadmium, arsenic, and mercury from the samples collected for this study, the results suggest that the mean concentrations of barium, cobalt, chromium, copper, lead, vanadium, cadmium, and mercury are 1.2-, 5.4-, 2.7-, 1.4-, 1.9-, 1.1-, 2.4-, and 2.6-times the background value of the local soil, respectively. This indicates that the dusts of the parks and squares in Baotou city were considerably polluted with these metals. Besides, the mean values of *I*_geo_ also depict the levels and order of pollution as Co > Cr > Cd > Hg > Pb > Cu > Ba > V > Ni > Mn > Zn > As. Thus, the dusts from the parks and squares of Baotou city were polluted by Cr, Co, Pb, Cd, and Hg significantly. From assessment of the outcomes of the Pollution Load Index (*PLI*), it is evident that the squares and parks of Baotou city were polluted by barium, cobalt, chromium, copper, manganese, nickel, lead, vanadium, zinc, cadmium, arsenic, and mercury in various degrees. Baogang Park and Baotou Paradise were revealed to be moderately polluted, while other investigated parks and squares showed to have been unpolluted to moderately polluted. The health risk analysis depicts that intake of the contaminated dust particles was the major pathway of exposure to the metals, especially for the children and the adults in the area, then through dermal contact. The adverse impacts of the dust particles to people’s health were also comparatively light in the Baotou city. Since children and adults are the most affected people, their health risks are higher when they get exposed to the dust particles containing the heavy metals discussed in the study. The present study also sought to determine the levels of contamination for the heavy metals and the health risks that they pose to people that are exposed to them. It was eminent that the finer particles (particle size < 100 μm), and even the finest among them (PM_10_ and PM_2.5_) from the dust samples, could simply be re-suspended in the air, thus, posing serious environmental effects as well as health risk to people. Therefore, within the scope of this study, concentration of heavy metals in the dust particles (even for finer particles of PM_100_, PM_10_, and PM_2.5_ in size) and their effects on people’s health should be investigated in-depth in further studies. Meanwhile, trace element biomonitoring in the blood, urine, and hair of local people will also be conducted in follow-up work.

## Figures and Tables

**Table 1:** Metal concentration in dust collected from urban square and park of Baotou city (mg/kg).

Metal	Ba	Co	Cr	Cu	Mn	Ni	Pb	V	Zn	Cd	As	Hg
Min	499.9	25.1	103.9	12.1	336.9	17.6	21.9	57.8	13.6	0.1	5.1	23.9
Max	845.8	112.4	379.8	53.9	1148.4	32.4	68.9	108.7	88.7	0.6	8.2	192.0
Mean	640.4	52.9	154.1	26.9	504.4	25.1	36.2	71.3	49.7	0.3	6.5	64.9
Median	636.3	48.0	142.2	24.7	465.0	24.7	35.5	70.4	45.8	0.3	6.5	51.3
SD	101.6	18.3	57.2	10.9	149.8	4.5	11.1	9.7	20.4	0.1	0.7	40.4
CV(%)	15.9	34.6	37.1	40.4	29.7	18.0	30.7	13.6	41.1	39.4	11.0	62.3
Skewness	0.34	1.58	2.92	0.56	3.40	0.05	1.05	2.41	0.18	0.90	0.10	1.83
Kurtosis	−0.96	3.62	9.98	−0.20	14.25	−0.98	1.52	8.45	−0.65	1.07	0.24	3.55
Reference value	527.7	9.93	56.39	19.17	508.6	24.5	18.76	65.38	55.68	0.1164	9.68	24.9

GM: Geometric Mean: Standard Deviation; CV: Coefficient of Variation; Hg: ng/g.

## Supplementary Materials

The following are available online at www.mdpi.com/1660-4601/14/8/886/s1, Table S1: Information from urban parks and squares of Baotou city; Table S2: Parameter meaning and value of daily dose model of heavy metals in urban surface dusts; Table S3: Exposure dose, hazard quotients, hazard indexes, and cancer risks of metals in park/square dust of Baotou.
