# Supplementary material for: Health Risks and Contamination Levels of Heavy Metals in Dusts from Parks and Squares of an Industrial City in Semi-Arid Area of China
Source: Int J Environ Res Public Health. 2017 Aug 7;14(8):886. doi: 10.3390/ijerph14080886 (PMC5580590; doi:10.3390/ijerph14080886)
Supplement: Supplementary file 1 [file ijerph-14-00886-s001.pdf]

International Journal of
Environmental Research and Public Health

MDPI

Supplementary Materials

Health Risks and Contamination Levels of Heavy Metals in Dusts from Parks and Squares of an Industrial City in Semi-Arid Area of China

Xiufeng Han, Xinwei Lu, Qinggeletu and Yongfu Wu

Table S1. Information from urban parks and squares of Baotou city.

Region	Park/Square	Region	Park/Square
Qingshan District	Music Square (L1)	Kundulun District	Aerding plant garden (L9)
	Jinglin park (L2)		Kundulun government Square (L10)
	Second Machinery Factory square (L3)		Bayi park (L11)
	First Machinery Factory square (L4)		Baogang park (L12)
	Dongfeng park (L5)		Baotou paradise (L13)
	Qingshan government square (L6)	Jiuyuan District	Cultural Square (L18)
	Children park (L7)		Bayingaole park (L19)
	Laodong park (L8)		Hatungaole park (L20)
	Jinxiu park (L14)		
	Saihantala park (L15)	Donghe District	Renmin park (L21)
	Nanjiao park (L16)		Cultural Square (L22)
	Wusutu park (L17)		Nangedong square (L23)
	Weapon Park (L25)		Government square (L24)
	Siji square (L26)		

Table S2. Parameter meaning and value of daily dose model of heavy metals in urban surface dusts.

Parameter	Meaning and Unit	Values	Reference
C	concentration of metal in dust, mg/kg	95%UCL	This study
IngR	ingestion rate, *mg/d*	200 (child), 100 (adults)	USEPA, 2001
InhR	inhalation rate, m^3/d	7.6(child), 20(adults)	Van den Berg, 1995
EF	exposure frequency, *d/y*	180	(USEPA, 2001
ED	exposure duration, *y*	6 (child), 24 (adults)	USEPA, 2001
SL	skin adherence factor, $mg/(cm^2h)$	0.2(child), 0.7 (adults)	USEPA, 2001
SA	exposed skin area, cm^2	2800 (child), 5700 (adults)	USEPA, 2001
ABS	dimensionless dermal absorption factor, unitless	0.001, 0.03 for As	Ferreira-Baptista and De Miguel, 2005
PEF	particle emission factor, m^3/kg	1.36×10^9	USEPA, 2001
BW	average body weight, *kg*	15 (child), 70 (adults)	USEPA, 1989
AT	averaging time, *d*	ED × 365 (for non-carcinogens) 70 × 365 (for carcinogens)	USEPA, 1989
VF	Thevolatilization factor ,m^3/kg		USEPA 2001

Table S3. Exposure dose, hazard quotients, hazard indexes and cancer risks of metals in park/square dust of Baotou.

Element	Ba	Co	Cr	Cu	Mn	Ni	Pb	V	Zn	Cd	As	Hg
C(95% UCL)	681	60.3	177	31.2	565	26.9	40.7	75.2	58.0	0.33	6.82	0.0812
RfD_{ing}	7.00×10^{-2}	2.00×10^{-2}	3.00×10^{-3}	4.00×10^{-2}	4.60×10^{-2}	2.00×10^{-2}	3.50×10^{-3}	7.00×10^{-3}	3.00×10^{-1}	1.00×10^{-3}	3.00×10^{-4}	3.00×10^{-4}
RfD_{inh}	1.43×10^{-4}	5.71×10^{-6}	2.86×10^{-5}	4.02×10^{-2}	1.43×10^{-5}	2.06×10^{-2}	3.52×10^{-3}	7.00×10^{-3}	3.00×10^{-1}	1.00×10^{-3}	3.01×10^{-4}	8.57×10^{-5}
RfD_{dermal}	4.90×10^{-3}	1.60×10^{-2}	6.00×10^{-5}	1.20×10^{-2}	1.84×10^{-3}	5.40×10^{-3}	5.25×10^{-4}	7.00×10^{-5}	6.00×10^{-2}	1.00×10^{-5}	1.23×10^{-4}	2.10×10^{-5}
Sf_{ing}											1.50	
Sf_{inh}		9.80	42.0			0.84				6.30	15.1	
Sf_{demal}											3.66	
Children												
D_{ing}	4.48×10^{-3}	3.97×10^{-4}	1.17×10^{-3}	2.05×10^{-4}	3.71×10^{-3}	1.77×10^{-4}	2.68×10^{-4}	4.95×10^{-4}	3.81×10^{-4}	2.17×10^{-6}	4.49×10^{-5}	5.34×10^{-7}
D_{inh}	1.25×10^{-7}	1.11×10^{-8}	3.26×10^{-8}	5.74×10^{-9}	1.04×10^{-7}	4.94×10^{-9}	7.48×10^{-9}	1.38×10^{-8}	1.07×10^{-8}	6.06×10^{-11}	1.25×10^{-9}	1.49×10^{-11}
D_{dermal}	1.25×10^{-5}	1.11×10^{-6}	3.26×10^{-6}	5.75×10^{-7}	1.04×10^{-5}	4.95×10^{-7}	7.49×10^{-7}	1.38×10^{-6}	1.07×10^{-6}	6.07×10^{-9}	3.77×10^{-6}	1.49×10^{-9}
D_{vapour}												6.21×10^{-7}
HQ_{ing}	6.40×10^{-2}	1.98×10^{-2}	3.88×10^{-1}	5.14×10^{-3}	8.07×10^{-2}	8.84×10^{-3}	7.65×10^{-2}	7.07×10^{-2}	1.27×10^{-3}	2.17×10^{-3}	1.50×10^{-1}	1.78×10^{-3}
HQ_{inh}	8.76×10^{-4}	1.94×10^{-3}	1.14×10^{-3}	1.43×10^{-7}	7.26×10^{-3}	2.40×10^{-7}	2.12×10^{-6}	1.97×10^{-6}	3.55×10^{-8}	6.06×10^{-8}	4.16×10^{-6}	1.74×10^{-7}
HQ_{dermal}	2.56×10^{-3}	6.94×10^{-5}	5.44×10^{-2}	4.79×10^{-5}	5.65×10^{-3}	9.17×10^{-5}	1.43×10^{-3}	1.98×10^{-2}	1.78×10^{-5}	6.07×10^{-4}	3.06×10^{-2}	7.12×10^{-5}
HQ_{vapour}												7.24×10^{-3}
$HI = \sum HQ$	6.74×10^{-2}	2.18×10^{-2}	4.44×10^{-1}	5.18×10^{-3}	9.37×10^{-2}	8.93×10^{-3}	7.79×10^{-2}	9.04×10^{-2}	1.29×10^{-3}	2.78×10^{-3}	1.80×10^{-1}	9.10×10^{-3}
Adults												
D_{ing}	4.80×10^{-4}	4.25×10^{-5}	1.25×10^{-4}	2.20×10^{-5}	3.98×10^{-4}	1.89×10^{-5}	2.87×10^{-5}	5.30×10^{-5}	4.09×10^{-5}	2.32×10^{-7}	4.81×10^{-6}	5.72×10^{-8}
D_{inh}	7.06×10^{-8}	6.25×10^{-9}	1.84×10^{-8}	3.24×10^{-9}	5.85×10^{-8}	2.79×10^{-9}	4.22×10^{-9}	7.79×10^{-9}	6.01×10^{-9}	3.42×10^{-11}	7.07×10^{-10}	8.41×10^{-9}
D_{dermal}	1.92×10^{-5}	1.70×10^{-6}	4.98×10^{-6}	8.78×10^{-7}	1.59×10^{-5}	7.56×10^{-7}	1.14×10^{-6}	2.11×10^{-6}	1.63×10^{-6}	9.27×10^{-9}	5.75×10^{-6}	2.28×10^{-6}
D_{vapour}												3.50×10^{-7}
HQ_{ing}	6.86×10^{-3}	2.12×10^{-3}	4.16×10^{-2}	5.50×10^{-4}	8.65×10^{-3}	9.47×10^{-4}	8.19×10^{-3}	7.57×10^{-3}	1.36×10^{-4}	2.32×10^{-4}	1.60×10^{-2}	1.91×10^{-4}
HQ_{inh}	4.94×10^{-4}	1.09×10^{-3}	6.42×10^{-4}	8.05×10^{-8}	4.09×10^{-3}	1.35×10^{-7}	1.20×10^{-6}	1.11×10^{-6}	2.00×10^{-8}	3.42×10^{-8}	2.35×10^{-6}	9.82×10^{-8}
HQ_{dermal}	3.91×10^{-3}	1.06×10^{-4}	8.30×10^{-2}	7.32×10^{-5}	8.63×10^{-3}	1.40×10^{-4}	2.18×10^{-3}	3.02×10^{-2}	2.72×10^{-5}	9.27×10^{-4}	4.68×10^{-2}	1.09×10^{-4}
HQ_{vapour}												4.09×10^{-3}
$HI = \sum HQ$	1.13×10^{-2}	3.32×10^{-3}	1.25×10^{-1}	6.24×10^{-4}	2.14×10^{-2}	1.09×10^{-3}	1.04×10^{-2}	3.78×10^{-2}	1.63×10^{-4}	1.16×10^{-3}	6.28×10^{-2}	4.09×10^{-3}
Cancer risk		3.03×10^{-8}	3.82×10^{-7}			1.16×10^{-9}				9.69×10^{-11}	1.75×10^{-8}	
